# Cytotoxicity of Amyloid β1–42 Fibrils to Brain Immune Cells

**DOI:** 10.1021/acschemneuro.4c00835

**Published:** 2025-03-08

**Authors:** Mikhail Matveyenka, Mikhail Sholukh, Dmitry Kurouski

**Affiliations:** † Department of Biochemistry and Biophysics, Texas A&M University, College Station, Texas 77843, United States; ‡ Department of Biology, Belarussian State University, Minsk 220030, Belarus

**Keywords:** amyloid β_1−42_, macrophages, dendritic cells, microglia, neurons

## Abstract

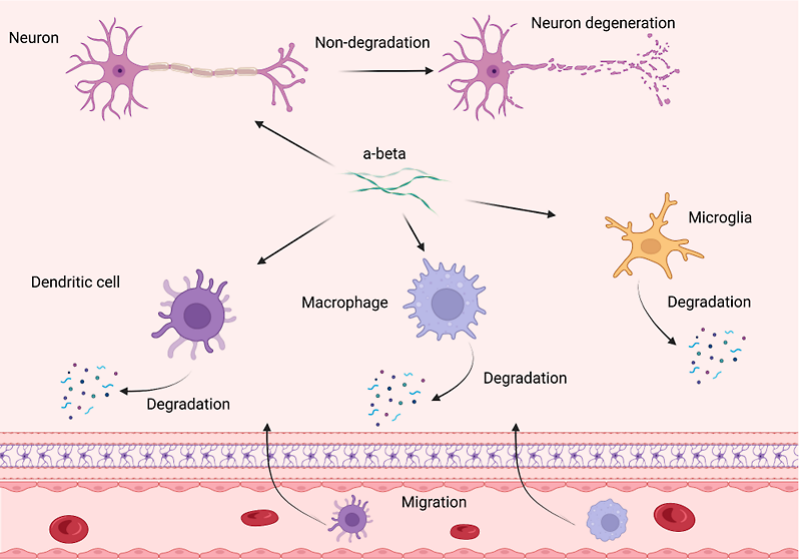

Alzheimer’s disease (AD) is a progressive pathology that is linked to abrupt aggregation of amyloid β_1–42_ (Aβ_1–42_) peptide in the central nervous system. Aβ_1–42_ aggregation yields amyloid oligomers and fibrils, toxic protein aggregates that cause progressive neuronal degeneration in the frontal lobe of the brain. Although neurons remain the focus of AD for decades, a growing body of evidence suggests that the degeneration of immune cells in the brain can be the major cause of AD. However, the extent to which Aβ_1–42_ aggregates are toxic to the major classes of immune cells in the brain remains unclear. In the current study, we examine the cytotoxic effects of Aβ_1–42_ fibrils on macrophages, dendritic cells, and microglia. These cells play vitally important roles in development and homeostasis of the central nervous system. We found that Aβ_1–42_ fibrils caused calcium release and enhanced levels of reactive oxygen species in macrophages, dendritic cells, and microglia as well as neurons. We also investigated the extent to which the lysozymes of these immune cells could alter the aggregation properties of Aβ_1–42_. Our results showed that lysosomes extracted from macrophages, dendritic cells, and microglia drastically accelerated Aβ_1–42_ aggregation as well as altered cytotoxicity of these protein aggregates. These results indicate that impairment of immune cells in the brain can be a critically important aspect of neurodegenerative processes that are taking place upon the onset of AD.

## Introduction

Macrophages, dendritic cells, and microglia are the key components of the brain’s immune system. Brain macrophages, also known as nonparenchymal macrophages, endocytose and digest viruses and microorganisms, as well as degrade apoptotic cells.^1^ Together with microglia, macrophages regulate brain development and maintain the homeostasis of the central nervous system. On the cellular level, microglia and macrophages can be differentiated based on the expressed transmembrane protein. Microglia cells express transmembrane protein 119 (TMEM119), P2Y purinoceptor 12 (P2RY12), and Sal-like protein 1 (SALL1). At the same time, nonparenchymal macrophages express CD45 and MHC class II molecules.^2^ Brain macrophages and microglia also interact with almost all cell types in the brain including dendritic cells.^3^ These cells also regulate the expression of mononuclear phagocytes.^2^ Dendritic cells exhibit similar macrophage and microglia activities. However, dendritic cells retain the endocytosed antigens and present them on MHC.^4^ Antigen presentation initiates T cell immune response, and other cascades are required for the activation of the immune system. Thus, macrophages, dendritic cells, and microglia are at the frontline of brain defense against viruses and microorganisms.

Several reported to date pieces of experimental evidence indicate that microglia directly and indirectly contributes to the onset and progression of neurodegenerative diseases in the brain.^5−7^ Another hallmark of such neurodegenerative diseases is the progressive aggregation of amyloidogenic proteins. Alzheimer’s disease (AD), for example, is linked to the abrupt aggregation of amyloid β_1–42_ (Aβ_1–42_) peptide.^8−10^ As a result, β-sheet-rich oligomers and fibrils are formed. Our group previously demonstrated that Aβ_1–42_ formed at least two types of oligomers with parallel and antiparallel β-sheets that later propagate into fibrils.^9^ We also demonstrated that toxicity of both oligomers and fibrils could be altered by phospholipids.^11^ Specifically, Aβ_1–42_ fibrils that were grown in the presence of cardiolipin and phosphatidylcholine exerted significantly stronger cell toxicity compared to Aβ_1–42_ aggregates formed in the lipid-free environment.^11^ Furthermore, these lipids uniquely altered the rate of Aβ_1–42_ aggregation. Zhaliazka and Kurouski recently reported that fatty acids could alter the cytotoxicity of Aβ_1–42_ fibrils.^8^ It was found that with an increase in the degree of unsaturation of fatty acids, the toxicity of Aβ_1–42_ fibrils increased.

Nevertheless, it remains unclear to what extent microglia, macrophages, and dendritic cells themselves are affected by amyloid aggregates. In the current study, we utilized a set of molecular methods to examine cytotoxicity of Aβ_1–42_ fibrils on microglia, macrophages, and dendritic cells, as well as on the ability of such aggregates to alter lysosomal activity of these cells. We found that amyloid fibrils strongly suppressed the lysosomal activity of immune cells, whereas this impairment was not observed for neurons. These results suggest that immune cells excrete protein aggregates via exocytosis. Expanding upon this, we used a set of biophysical methods to investigate the extent to which lysosomes extracted from microglia, macrophages, and dendritic cells altered Aβ_1–42_ aggregation. We found that lysosomes from different immune cells uniquely alter the aggregation rate of Aβ_1–42_, as well as modified the morphology and toxicity of Aβ_1–42_ fibrils. These results indicate that immune cells can play a very important role in the onset and spread of AD.

## Results and Discussion

**Figure 1:**
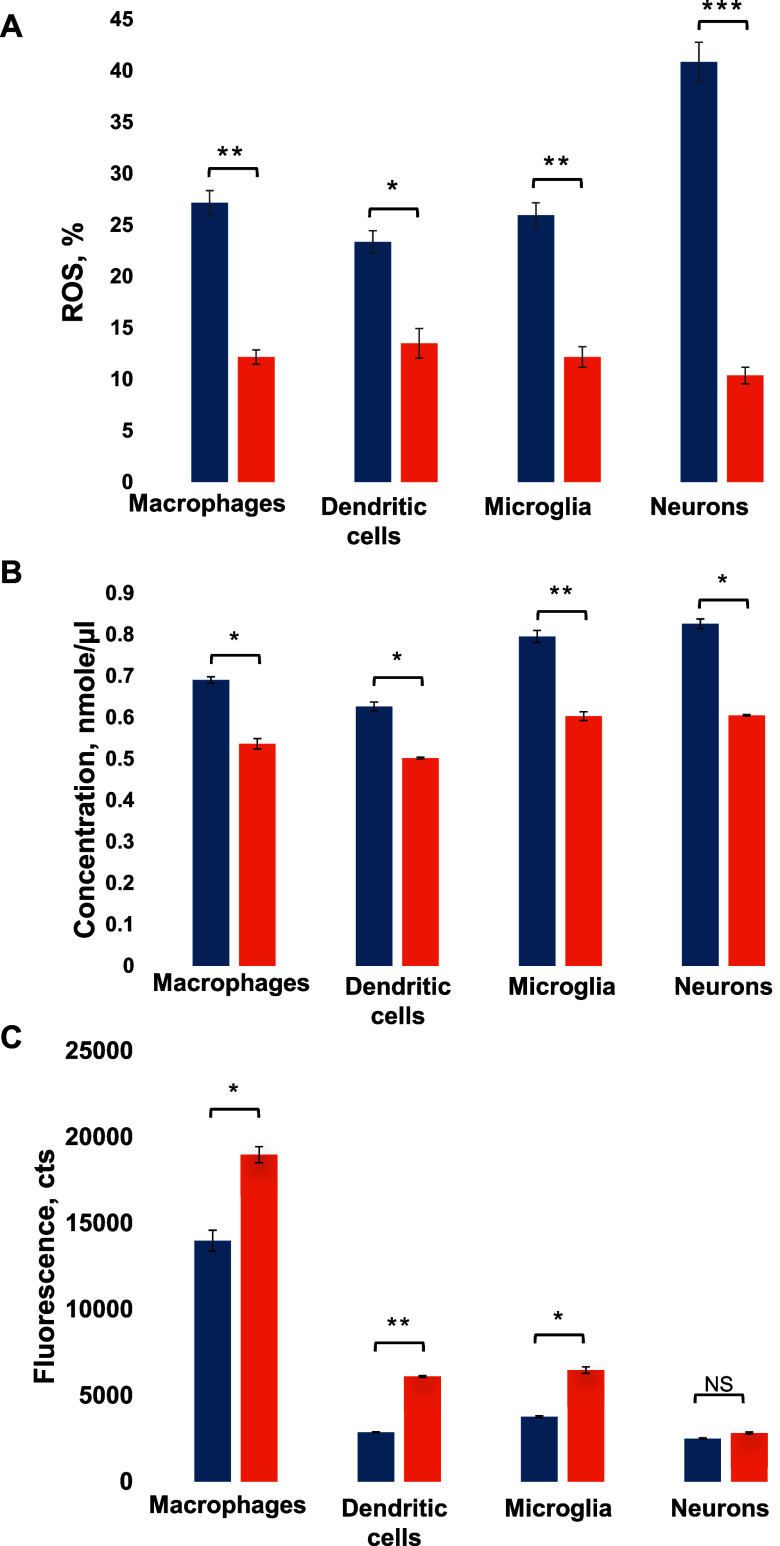
Aβ_1–42_ fibrils are toxic to macrophages, dendritic cells, microglia, and neurons. Histograms of ROS assays (A), Ca^2+^ release (B), and lysosomal activity (C) of macrophages, dendritic cells, microglia, and neurons exposed to Aβ_1–42_ fibrils (blue) and control (orange) cells. According to *t*-test, *P < 0.05, **P < 0.01, NS is nonsignificant differences.

First, we investigated the extent to which Aβ_1–42_ fibrils are toxic to macrophages, dendritic cells, microglia, and neurons. Using the ROS assay, we found that Aβ_1–42_ aggregates caused an increase in ROS levels in all cell types, Figure 1A. However, a substantially greater increase in the ROS levels was observed in neurons exposed to Aβ_1–42_ fibrils compared to the immune cells. These results indicate that Aβ_1–42_ fibrils are more toxic to neurons compared with immune cells. Similar results were obtained using calcium (Ca^2+^) release assay, Figure 1B. We found that Aβ_1–42_ fibrils triggered a similar magnitude of Ca^2+^ release in macrophages and dendritic cells, whereas the magnitude of Ca^2+^ release in microglia cells and neurons was slightly higher.

Next, we investigated whether Aβ_1–42_ fibrils could alter the lysosomal activity of immune cells. Our results indicate that Aβ_1–42_ aggregates caused a substantial decrease in the lysosomal activity in macrophages, dendritic cells, and microglia, while no changes in the lysosomal activity were observed in neurons, Figure 1C. These results indicate that immune cells actively engage lysosomes to clear out amyloid aggregates.

**Figure 2:**
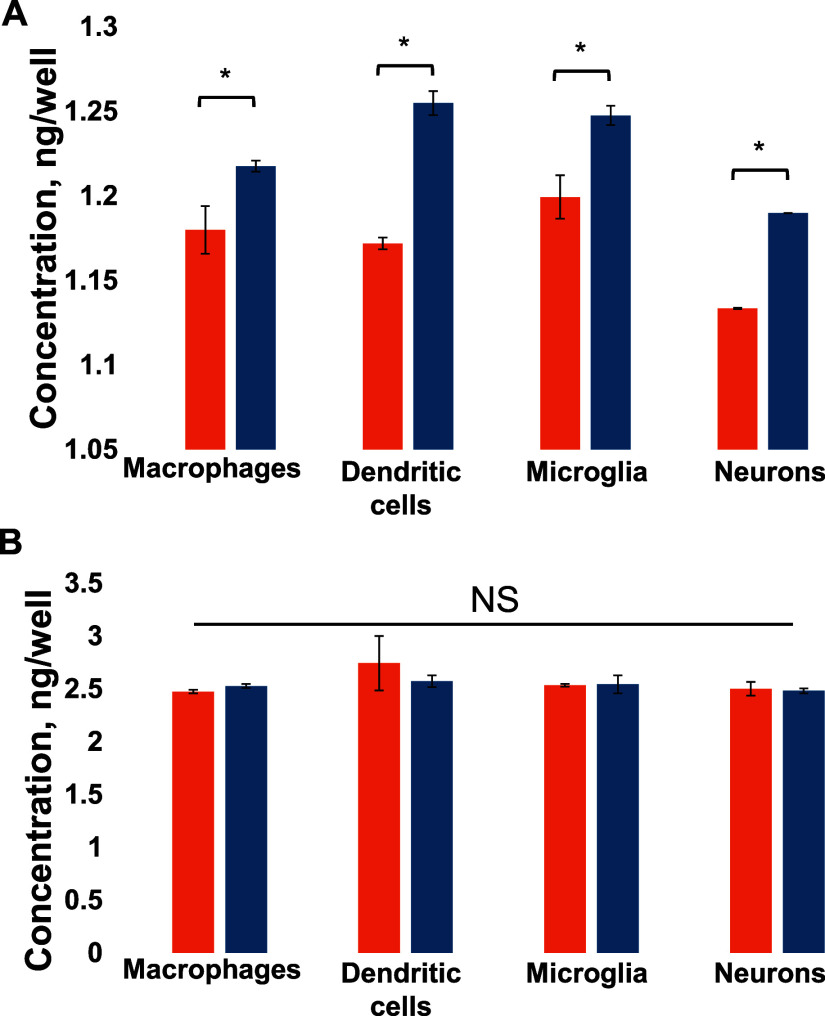
Aβ_1–42_ fibrils alter the expression of IL-1β in the immune cells and neurons. Histograms of expression of IL-1β (A) and IL-18 (B) in macrophages, dendritic cells, microglia, and neurons exposed to Aβ_1–42_ fibrils formed in the presence of corresponding lysosomes (blue) and control (orange) cells. According to *t*-test, *P < 0.05, NS is nonsignificant differences.

Previously, our group demonstrated that in response to contact with α-synuclein fibrils, macrophages release cytokines and chemokines, including interleukin-1 beta (IL-1β) and interleukin 18 (IL-18), to initiate the innate immune response.^12−14^ The release of these cytokines initiates a potent defensive inflammatory response.^15,16^ Expanding upon this, we investigated whether the contact of immune cells and neurons with Aβ_1–42_ fibrils can alter the release of IL-1β and IL-18. We found that all cell types increased the release of IL-1β, while no changes in the expression of IL-18 were observed, as shown in Figure 2.

**Figure 3:**
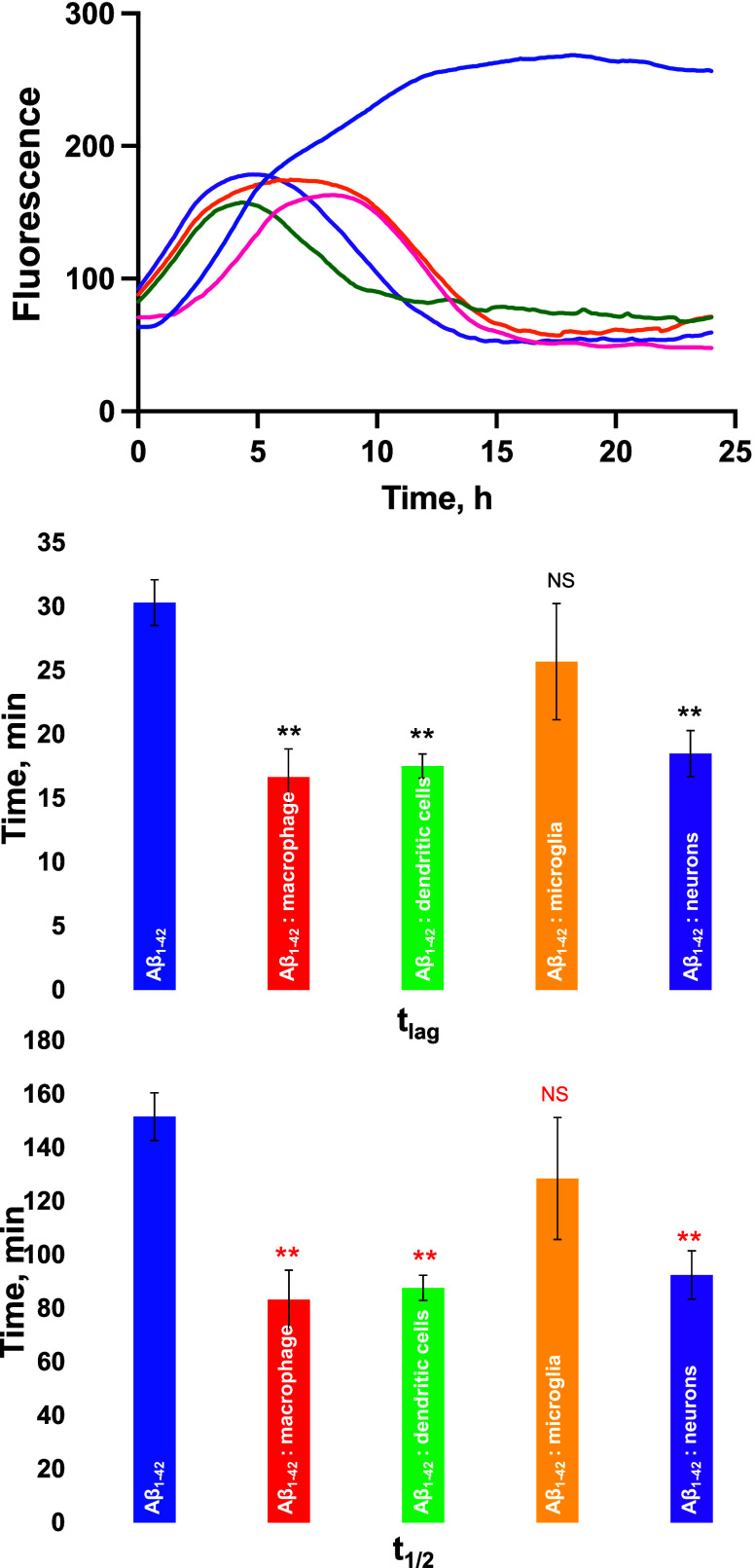
Lysosomes alter the aggregation rate of Aβ_1–42_. ThT curves of Aβ_1–42_ aggregation in the lipid-free environment (blue) as well as in the presence of lysosomes extracted from macrophages (red), dendritic cells (green), microglia (orange), and neurons (purple) and histograms of *t*_lag_ (middle) and *t*_1/2_ (bottom). According to one-way ANOVA, **P < 0.01, NS is nonsignificant differences.

Lysosomal clearance of amyloid aggregates and changes in the expression of IL-1β indicate that immune cells accumulate Aβ_1–42_ in their lysosomes. Expanding upon this, we investigated whether lysosomes could alter the aggregation of Aβ_1–42_. To test this hypothesis, we incubated Aβ_1–42_ at 37 °C in the presence and absence of lysosomes that were extracted from macrophages, dendritic cells, microglia, and neurons. Using the thioflavin T (ThT) assay, we examined the extent to which lysosomes could alter the rate of Aβ_1–42_ aggregation. We found that in the lysosome-free environment, Aβ_1–42_ aggregates exhibit a lag phase (*t*_lag_) of ∼30 min that is followed by a rapid increase in ThT fluorescence, Figure 3. Such an increase indicates the formation of amyloid aggregates with a *t*_1/2_ of 150 min. Similar t_lag_ and *t*_1/2_ values were observed for Aβ_1–42_ in the presence of microglia lysosomes. However, lysosomes extracted from macrophages, dendritic cells, and neurons fully eliminated the lag phase of protein aggregation causing nearly instantaneous aggregation of Aβ_1–42_. These results indicate that lysosomes can drastically shorten the lag phase of Aβ_1–42_ aggregation and accelerate fibril formation. We also observed that after reaching the plateau, ThT curves of Aβ_1–42_ aggregation in the presence of lysosomes that were extracted from macrophages, dendritic cells, microglia, and neurons exhibited a decrease in fluorescence intensity. These results suggest that Aβ_1–42_ fibrils that were formed in the presence of lipid vesicles could have residual lipids on their surfaces, which, in turn, triggered their precipitation. As a result, less fibril aggregates would be present in the solution and, consequently, its fluorescence intensity will decrease.

**Figure 4:**
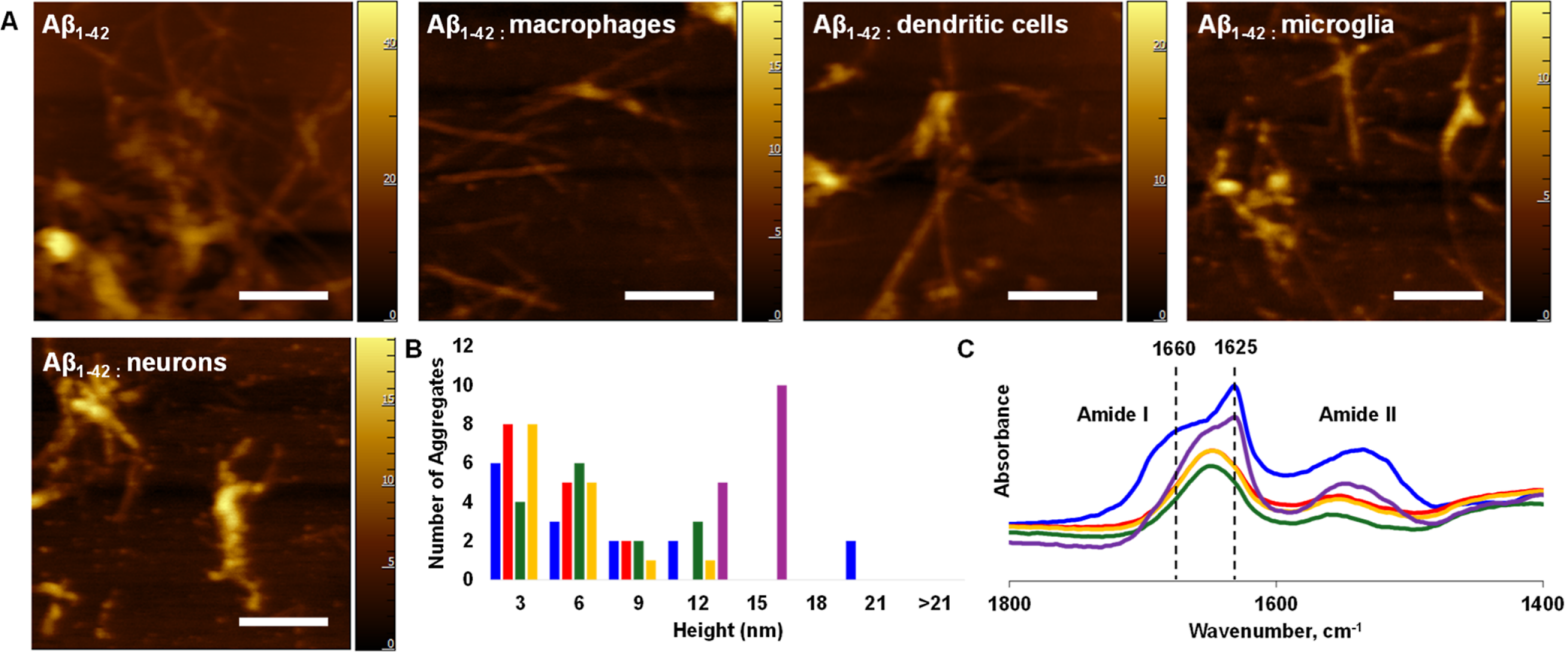
Lysosomes change the morphology and secondary structure of Aβ_1–42_ aggregates. AFM (A) images with corresponding height profiles (B) and FTIR spectra (C) of Aβ_1–42_ fibrils formed in the absence of lipids and in the presence of lysosomes extracted from macrophages, dendritic cells, microglia, and neurons.

Next, we utilized atomic force microscopy (AFM) to examine the morphology of Aβ_1–42_ aggregates formed in the presence and absence of lysosomes that were extracted from macrophages, dendritic cells, microglia, and neurons, as shown in Figure 4. We observed morphologically similar fibrils in all analyzed samples with heights ranging from 3 to 12 nm. However, Aβ_1–42_ fibrils that were grown in the presence of neuronal lysosomes had significantly larger heights compared to those of all other protein aggregates. Using infrared (IR) spectroscopy, we also investigated the extent to which the presence of lysosomes altered the secondary structure of Aβ_1–42_ fibrils. IR spectra acquired from Aβ_1–42_ fibrils grown in the absence of lysosomes and in the presence of lysosomes extracted from neurons exhibit amide I at 1625 cm^–1^ with a shoulder around 1660 cm^–1^. These spectroscopic signatures indicate the predominance of parallel β-sheets in the secondary structures of these fibrils with a small amount of unordered protein. At the same time, IR spectra acquired from Aβ_1–42_ fibrils grown in the presence of lysosomes extracted from immune cells exhibit amide I centered at 1645 cm^–1^, which corresponds to α-helix. These results indicate that lysosomes uniquely alter the morphology and secondary structure of Aβ_1–42_ aggregates formed in their presence.

**Figure 5:**
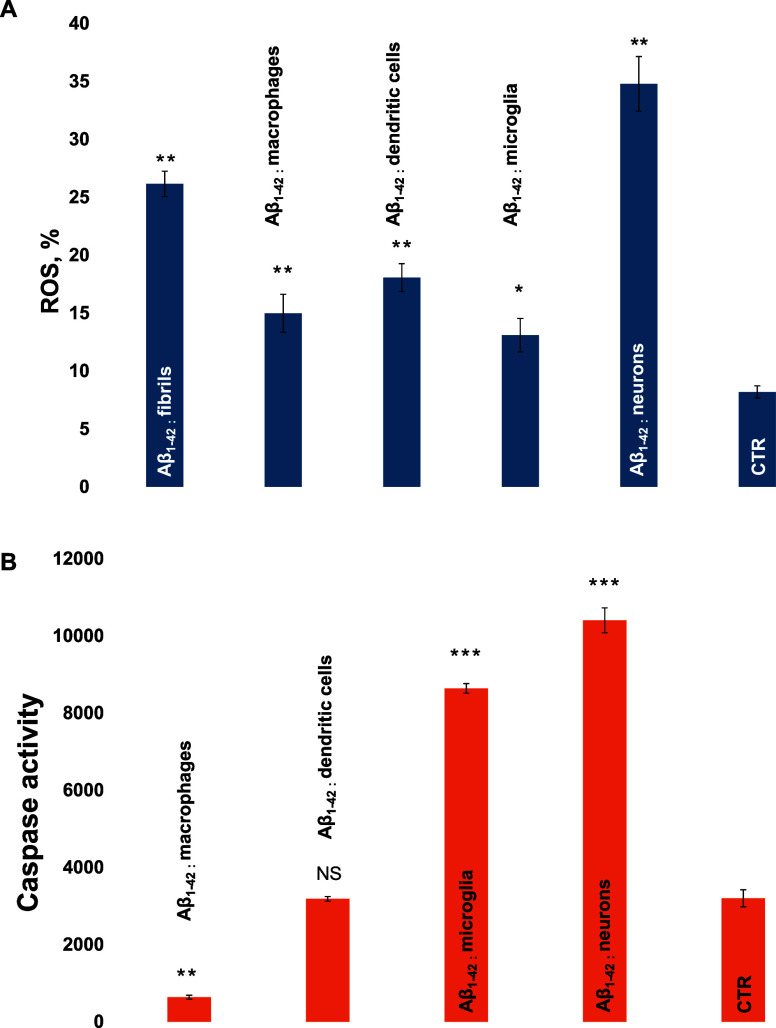
Lysosomes alter toxicity of Aβ_1–42_ fibrils to neurons. Histograms of ROS (A) and caspase-1 (B) assay in neurons exposed to Aβ_1–42_ fibrils formed in the absence of lipids and in the presence of lysosomes extracted from macrophages, dendritic cells, microglia, and neurons exposed. According to ANOVA, *P < 0.05, **P < 0.01, NS is nonsignificant differences.

One can expect that Aβ_1–42_ aggregates formed in macrophages, dendritic cells, microglia, and neurons would be exocytosed by these cells. In this case, they can be up taken by neurons. Expanding upon this, we investigated the extent to which such protein aggregates could be toxic to neurons. ROS assay showed that Aβ_1–42_ aggregates formed in the presence of neuronal lysosomes exerted substantially higher cytotoxicity than Aβ_1–42_ aggregates grown in the absence of lysosomes, Figure 5. At the same time, Aβ_1–42_ fibrils grown in the presence of lysosomes extracted from macrophages, dendritic cells, and microglia appeared to be significantly less toxic to neurons than Aβ_1–42_ aggregates grown in the absence of lysosomes.

### Similar Results Were Obtained Using Caspase-1 Assay

Specifically, we found that Aβ_1–42_ fibrils grown in the presence of lysosomes extracted from macrophages and dendritic cells were not toxic to neurons, whereas Aβ_1–42_ fibrils grown in the presence of lysosomes extracted from microglia and neurons caused a strong upregulation of caspase-1 in neurons. Finally, our results indicate that Aβ_1–42_ fibrils grown in the presence of lysosomes extracted from all cells increased the expression of IL-1β in neurons, while an increase in the expression of IL-18 was observed only as a result of neurons exposition to Aβ_1–42_ fibrils grown in the presence of lysosomes extracted from microglia and neurons, as well as Aβ_1–42_ fibrils formed in the lipid-free environment, Figure S1.

## Conclusions

Our results show that Aβ_1–42_ fibrils are toxic to neurons and brain immune cells, including macrophages, dendritic cells, and microglia. Endocytosis of such aggregates increases ROS levels in the cells, which ultimately causes their death. At the same time, unlike neurons, macrophages, dendritic cells, and microglia accumulate Aβ_1–42_ in their lysosomes. Biophysical assays used in our study indicate that lysosomes accelerate the aggregation of Aβ_1–42_ and change the morphology of these protein aggregates compared to Aβ_1–42_ fibrils formed in the lipid-free environment. Furthermore, Aβ_1–42_ fibrils formed in the presence of macrophages, dendritic cells, and microglia lysosomes exert lower cytotoxicity to neurons compared to Aβ_1–42_ fibrils formed in the lipid-free environment. These results indicate that immune cells can be involved in the suppression of cytotoxic effects of Aβ_1–42_ fibrils formed in the brain. We also found that Aβ_1–42_ aggregation in the presence of neuronal lysosomes strongly increased the cytotoxicity of Aβ_1–42_ fibrils. These results indicate that lysosomal activity of neurons, as well as their endo- and exocytosis activity, can be responsible for the spread of Aβ_1–42_ aggregates and, consequently, progression of AD.

## Experimental Section

### Materials

**Aβ**_**1**–**42**_ was purchased from GenScript, USA.

## Lysosome Extraction

Cells were homogenized using a tissue grinder. The homogenate was centrifuged at 500*g* for 10 min to separate cellular components. The supernatant was layered onto a discontinuous density gradient to separate cellular organelles based on their densities. A band containing the enriched lysosome fraction from the gradient was removed. The enriched lysosome fraction was diluted with PBS and centrifuged at 18,000*g* for 30 min to pellet the purified lysosomes. Extracted lysosomes were quantified using LAMP-1 Polyclonal Antibody (Cat #BS-1970R), Figure S2, and dynamic light scattering (Figures S3 and S4 and Table S1). Concentration of protein in the extracted lysosomes was characterized using a Bradford assay (Table S2).

### Protein Aggregation

To perform protein aggregation in a lipid-free environment, **Aβ**_**1**–**42**_ was dissolved in hexafluoro-2-propanol (HFIP), after which HFIP was evaporated, and the residue was dissolved in PBS to reach the final protein concentration of 40 μM. The protein was mixed with lysosomes at a 1:2 ratio. Next, samples were placed into a 96-well plate that was kept in the plate reader (Tecan, Männedorf, Switzerland) at 37 °C for 24 h under 120 rpm agitation.

### Kinetic Measurements

Rates of **Aβ**_**1**–**42**_ aggregation with and without lysosomes were measured using a ThT fluorescence assay. For this, samples were mixed with 2 mM of ThT solution and placed into the 96-well plate that was kept in the plate reader (Tecan, Männedorf, Switzerland) at 37 °C for 24 h under 120 rpm agitation. Fluorescence measurements were taken every 5 min (excitation 450 nm; emission 488 nm).

### AFM Imaging

Microscopic analysis of α-Syn aggregates was made on an AIST-NT-HORIBA system (Edison, NJ). Silicon AFM probes with a force constant of 2.7 N/m and resonance frequency of 50–80 kHz were used for all sample imaging. The probes were purchased from AppNano (Mountain View, CA, USA). Preprocessing of the collected AFM images was done using AIST-NT software (Edison, NJ, USA).

### Attenuated Total Reflectance Fourier-Transform Infrared Spectroscopy

An aliquot of the protein sample was placed onto the ATR crystal and dried at room temperature. Spectra were measured using a Spectrum 100 FTIR spectrometer (PerkinElmer, Waltham, MA, USA). Three spectra were collected from each sample and averaged using Thermo GRAMS Suite software (Thermo Fisher Scientific, Waltham, MA, USA).

### Cell Culturing

Rat midbrain N27 neuronal cells were purchased from ATCC and grown in RPMI 1640 Medium (Thermo Fisher Scientific, Waltham, MA, USA) with 10% heat-inactivated fetal bovine serum (FBS) (Invitrogen, Waltham, MA, USA) in a 96-well plate (30,000 cells per well) at 37 °C and 5% CO_2_. Macrophages H36.12j [Pixie 12j] were purchased from ATCC and grown in RPMI-1640 (Thermo Fisher Scientific, Waltham, MA, USA) with 10% heat-inactivated FBS (Invitrogen, Waltham, MA, USA) in a 96-well plate (30,000 cells per well) at 37 °C and 5% CO_2_. Dendritic cells (DC2.4) were purchased from ATCC and RPMI-1640 with 10% heat-inactivated FBS (Invitrogen, Waltham, MA, USA) in a 96-well plate (30,000 cells per well) at 37 °C and 5% CO_2_. Microglia cells (SIM-A9) were purchased from ATCC and grown in a mix of medium DMEM and F12 in ratio 1:1 and 10% heat-inactivated FBS (Invitrogen, Waltham, MA, USA) and 5% heat-inactivated horse serum (HS) (Invitrogen, Waltham, MA, USA) in a 96-well plate (30,000 cells per well) at 37 °C and 5% CO_2_. Antibiotic Normocin (InvivoGen, CA, USA) was added to all cell cultures to prevent bacterial contaminations.

After 24 h of incubation with the sample of **Aβ**_**1**–**42**_ aggregates, the cells were stained with annexin V and analyzed on an LSRII BD flow cytometer (BD, San Jose, CA). All measurements were made in triplicate, Figure S5.

### Expression of Cytokines

Cell samples were collected after incubation with the protein samples. High-binding plates were coated with primary antibodies at a concentration of 2 μg/mL in a volume of 50 μL. The plates were incubated for 2 h at room temperature. The plates were washed 3 times with a wash buffer. To reduce nonspecific antibody binding, the plates were incubated for 2 h at room temperature with 300 μL of 1× blocking buffer. The plates were then washed again with wash buffer. Samples in 1× blocking buffer were diluted 2-fold. 50 μL of standards and samples were added to the wells and incubated for 2 h at room temperature with shaking at 400 rpm. The wells were washed 3 times with 1× wash buffer. The diluted detection antibodies were prepared to a concentration of 0.5 μg/mL in 1× blocking buffer. 50 μL was added to each well and incubated for 1 h at room temperature with shaking at 400 rpm. The wells were washed 3 times with 1× wash buffer. The HRP-streptavidin solution was diluted to 0.05 μg/mL in 1× blocking buffer, and 50 μL was added to each well. The plates were incubated for 1 h while shaking at 400 rpm. 100 μL of TMB substrate was added to each well, and the plates were incubated for up to 20 min in the dark with shaking at 400 rpm. The reaction was stopped by adding 100 μL of the stop solution. Absorbance was measured in a plate reader at a wavelength of 450 nm.

## Supporting Information Available

Histogram of ELISA of IL-1β and IL-18; biophysical characteristics of extracted lysosomes; and histograms of cell viability according to annexin V assay (PDF)
