# Supplementary material for: Cytotoxicity of Amyloid β1–42 Fibrils to Brain Immune Cells
Source: ACS Chem Neurosci. 2025 Mar 8;16(6):1144–9. doi: 10.1021/acschemneuro.4c00835 (PMC11926782; doi:10.1021/acschemneuro.4c00835)
Supplement: Supplementary file 1 — cn4c00835_si_001.pdf [file cn4c00835_si_001.pdf]

Cytotoxicity of Amyloid β1-42 Fibrils to Brain Immune Cells

Mikhail Matveyenka[1], Mikhail Sholukh[2] and Dmitry Kurouski[*1]

1. Department of Biochemistry and Biophysics, Texas A&M University, College Station, Texas 77843, United States
2. Department of Biology, Belarussian State University, Minsk, 222000, Belarus

Supporting Information

Table S1. Radii of extracted lysosomes according to dynamic light scattering.

		Radius (nm)	Intensity (Cnt/s)	%PD	Particle Concentration (1/mL)
**Marcophages**	Mean	134	845479	38	6.78e+08
	S	6.2	49851	15.3	7.61e+07
	%S	4.7	6	40.2	1.12e+01
	S²	39	2485163853	232.8	5.79e+15
**Dendeitic cells**	Mean	262.9	1806815	54.2	6.14E+07
	S	319.5	699798	6.2	6.97E+06
	%S	121.5	39	11.4	1.14E+01
	S²	102051	4.89718E+11	38.3	4.86E+13
**Microglia**	Mean	143.9	1074211	52.6	1.13E+09
	S	13.3	125408	10.7	1.34E+08
	%S	9.3	12	20.4	1.18E+01
	S²	177.1	15727219013	114.8	1.78E+16
**Neurons**	Mean	143.1	2177143	52.5	1.00E+08
	S	9.5	486997	8.8	1.25E+07
	%S	6.6	22	16.8	1.25E+01

S²	89.8	2.37166E+11	77.5	1.56E+14

Table S2. Cocnentration of protein in extracted lysosomes according to Bradford assay.

**Lysosomes**	**mg/mL**
Macrophages	7.211
Dendritic cells	9.981
Microglia	13.974
Neurons	12.784

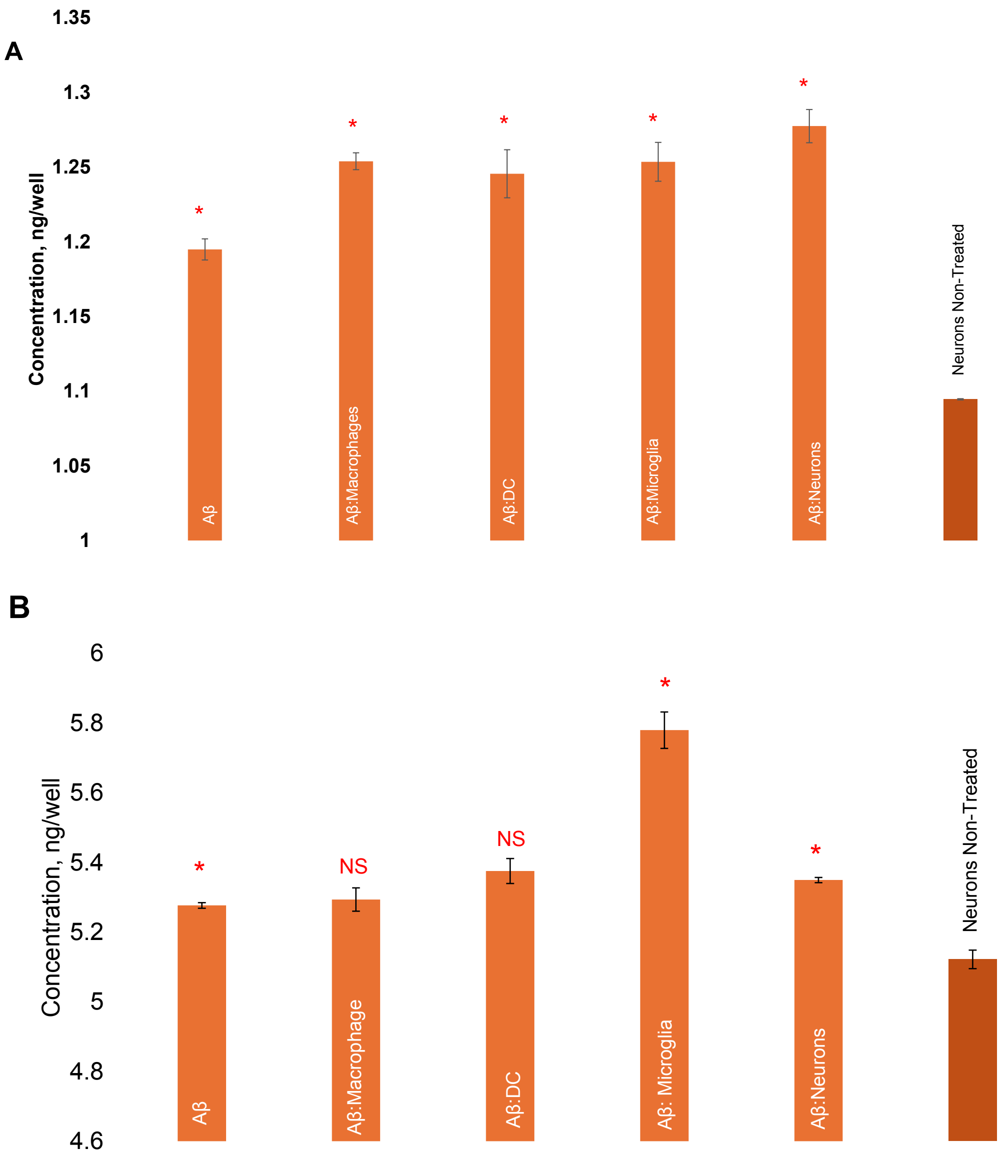

Figure S1. Histogram of ELISA of IL-1β (A) and IL-18 (B) expressed by neurons exposed to Aβ fibrils formed in the absence of lysosomes and in the presence of lysosomes extracted from macrophages, DC, microglia and neurons. According to one-way ANOVA, *$P<0.05$. NS is non-significant difference.

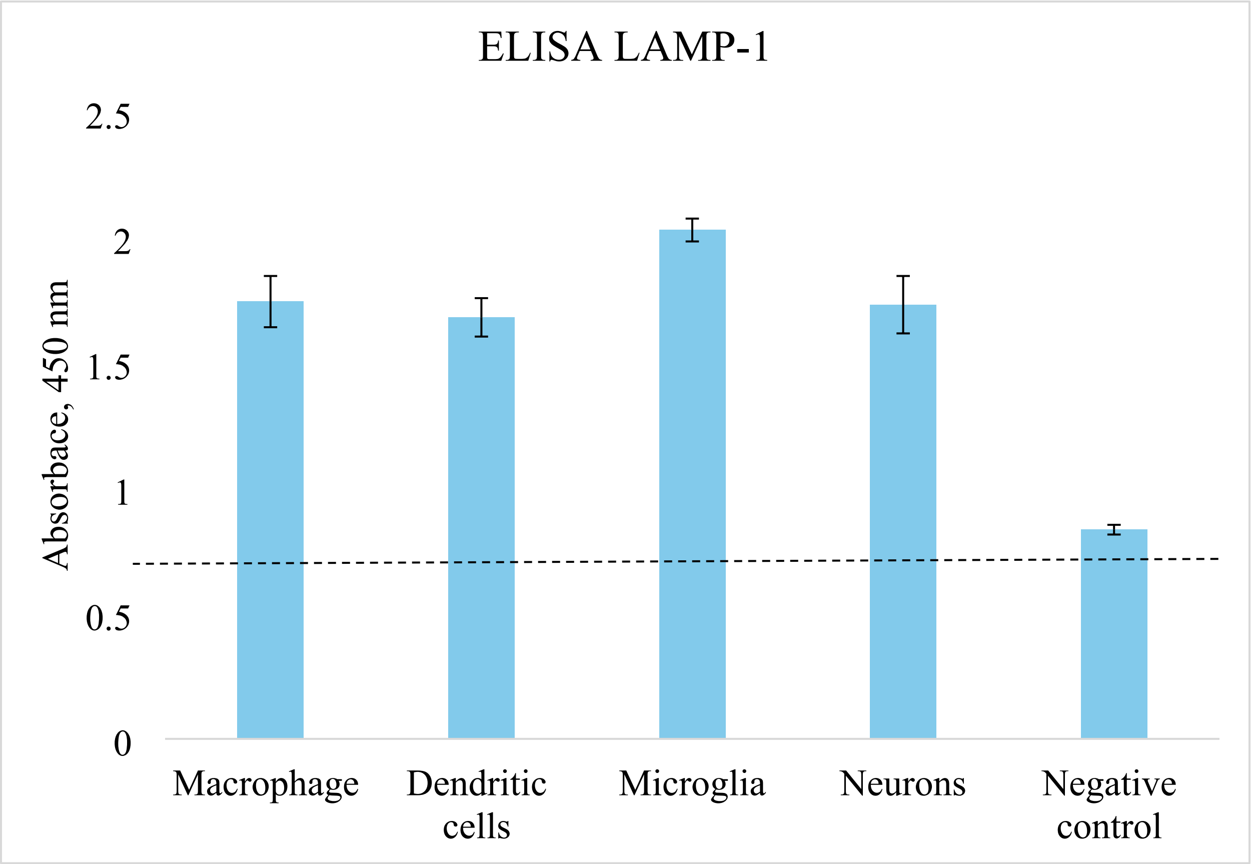

Figure S2. Histogram of absorbance intensities of LAMP-1 Polyclonal Antibody (Cat #BS-1970R) of extracted lysosomes from macrophages, dendritic cells, microglia and neurons.

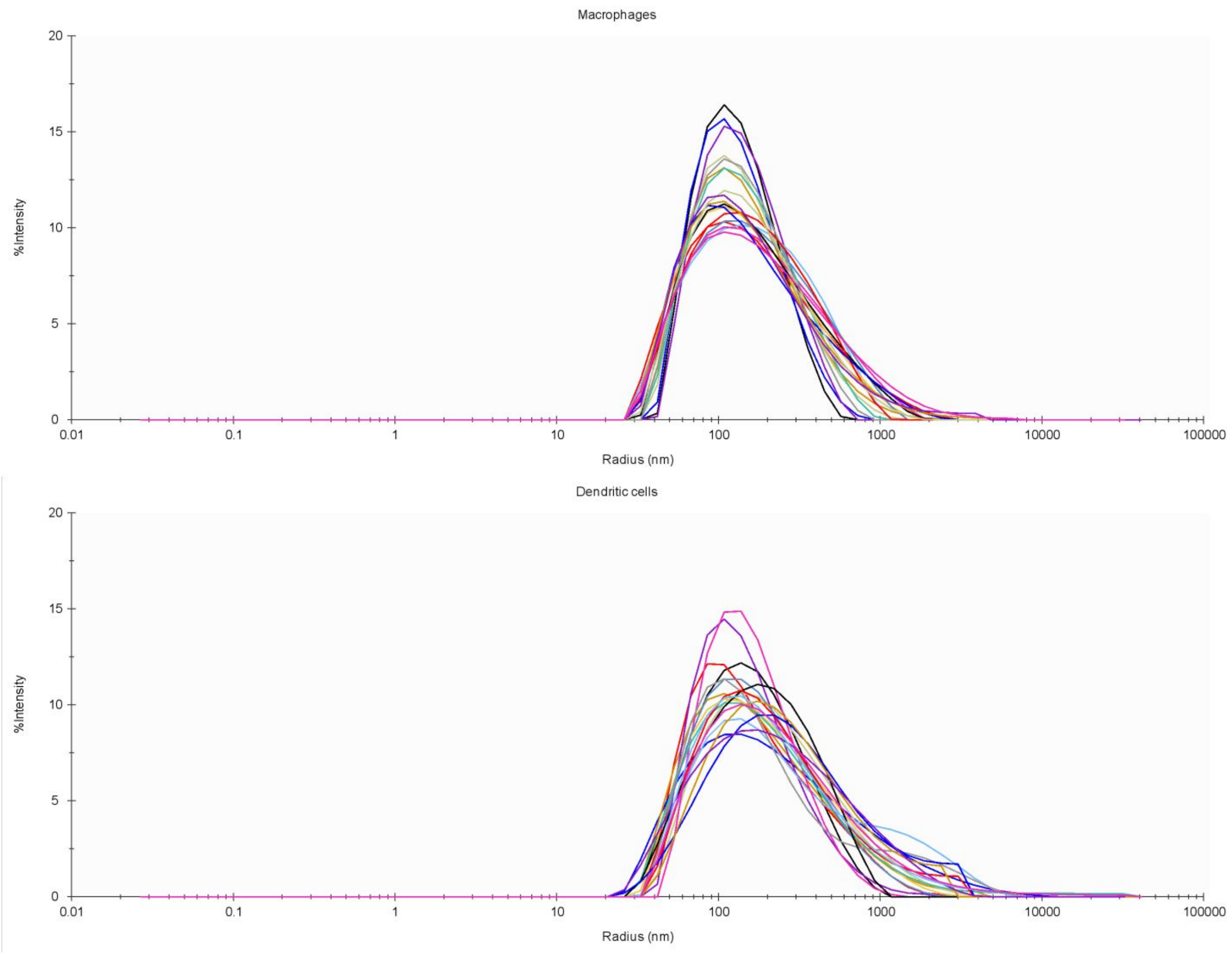

Figure S3. Dynamic light scattering analysis of extracted lysosomes from macrophages and dendritic cells.

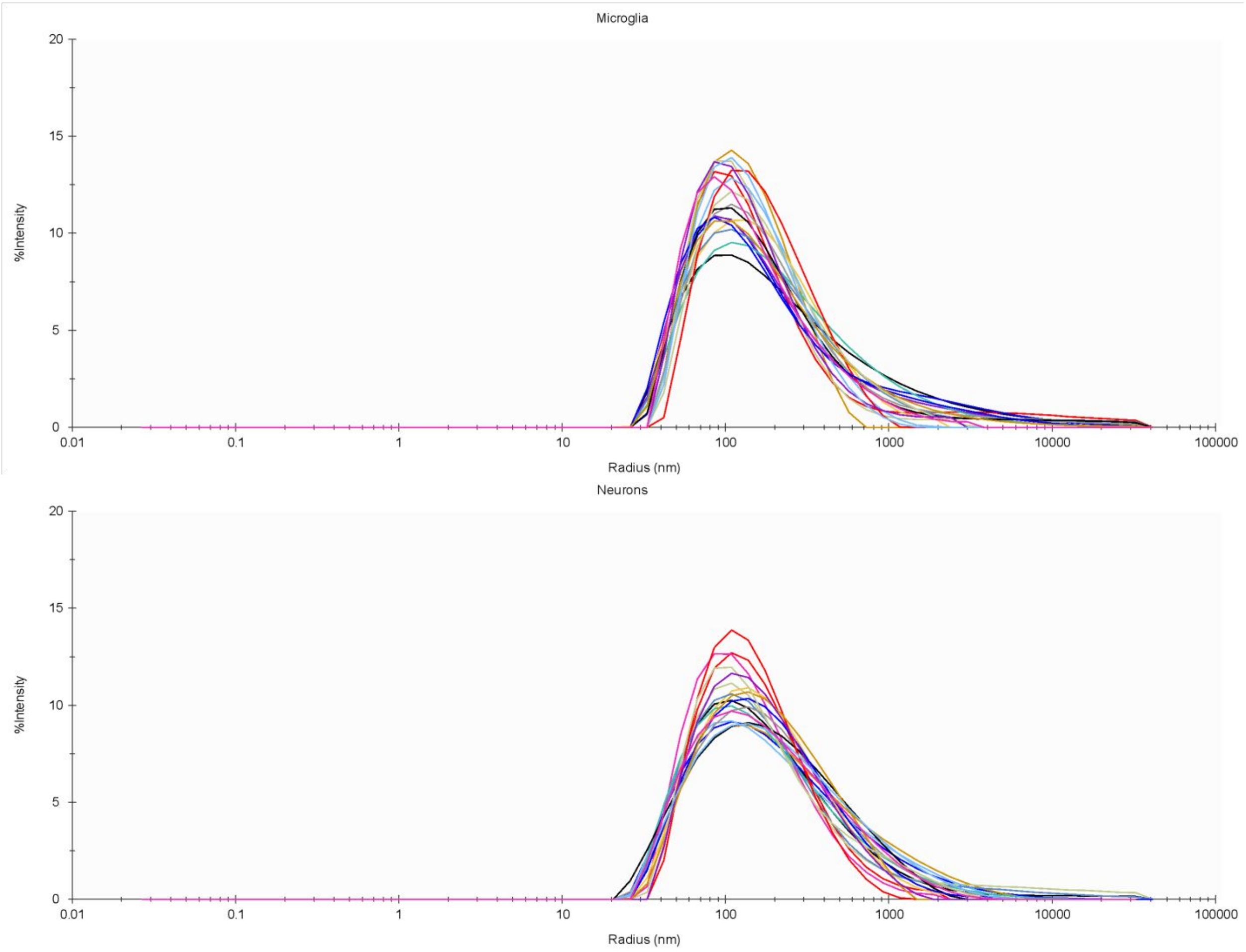

Figure S4. Dynamic light scattering analysis of extracted lysosomes from microglia and neurons.

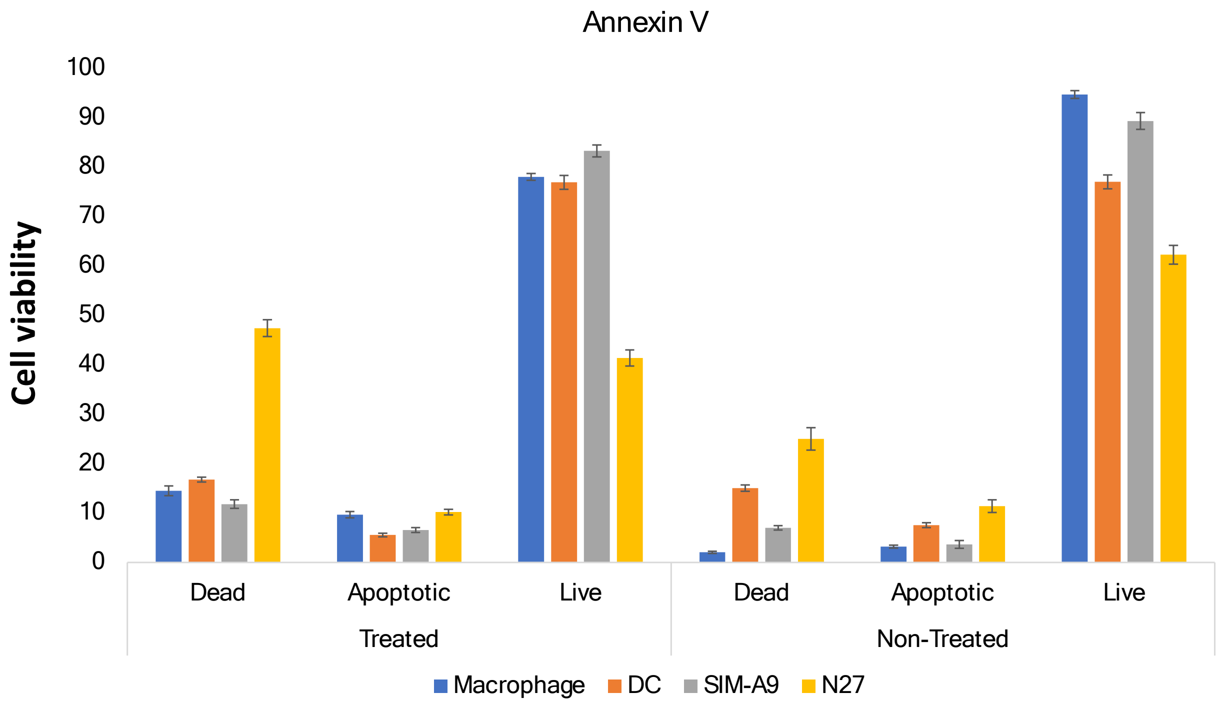

Figure S5. Histograms of cell viability according to annexin V assay.
